# On the Use of Dolomite as a Mineral Filler and Co-Filler in the Field of Polymer Composites: A Review

**DOI:** 10.3390/polym14142843

**Published:** 2022-07-13

**Authors:** Asfa Amalia Ahmad Fauzi, Azlin Fazlina Osman, Awad A. Alrashdi, Zaleha Mustafa, Khairul Anwar Abdul Halim

**Affiliations:** 1 Faculty of Chemical Engineering Technology, Universiti Malaysia Perlis (UniMAP), Arau 02600, Malaysia; asfauzi18@gmail.com (A.A.A.F.); kanwar@unimap.edu.my (K.A.A.H.); 2 Biomedical and Nanotechnology Research Group, Center of Excellent Geopolymer and Green Technology (CEGeoTech), Universiti Malaysia Perlis (UniMAP), Arau 02600, Malaysia; 3 Chemistry Department, Umm Al-Qura University, Al-Qunfudah University College, Al-Qunfudah Center for Scientific Research (QCSR), Al Qunfudah 21962, Saudi Arabia; aarashdi@uqu.edu.sa; 4 Faculty of Manufacturing Engineering, Universiti Teknikal Malaysia Melaka, Hang Tuah Jaya, Durian Tunggal 76100, Malaysia; zaleha@utem.edu.my

**Keywords:** dolomite, polymer composite, chemical and physical treatment, mechanical properties, hybrid filler

## Abstract

Polymers are being used in many applications all around the world. However, there are some drawbacks in the properties of polymers that could hamper their usage in certain applications. Therefore, a new material polymer composite was introduced. A polymer composite is a polymer-based material with the addition of a filler. Many researchers have reported the improvement in the properties of a polymer when a filler was introduced. This helps minimize the disadvantages of using a polymer. As a result, polymer composite products can be used in many industries, such as automobile, aerospace, biomedical, and packaging. Fillers derived from natural minerals, such as dolomite, are among the best reinforcement materials for polymeric materials because they are plentiful and low cost, have high rigidity and hardness, and even have tailorable surface chemistry. The use of dolomite as a filler in a polymer composite system has gained increasing attention in recent years after researchers successfully proved that it is capable of improving the mechanical, physical, and thermal properties of various polymeric materials. However, chemical or physical treatment/modification of raw dolomite is needed in order to prepare it as an efficient reinforcing filler. This procedure helps to improve the performance of the resultant polymer composites. This article reviews the usage of dolomite as a filler in a variety of polymeric materials and how it improved the performance of the polymer composite materials. It also highlights several methods that have been used for the purpose dolomite’s treatment/modification. Furthermore, the role of dolomite as a co-filler or a hybrid filler in a polymer composite system is also discussed, revealing the great potential and prospect of this mineral filler in the field of polymer composites for advanced applications.

## 1. Introduction

Today’s technological advancements have resulted in a variety of polymer composite materials being used in many different industries, including biomedical, concrete, paint, automotive, aerospace, marine, and food packaging industry [1,2,3,4]. The main reason for this is that their properties can be tailored to suit the requirements of specific applications by varying factors, such as the type of polymer, the filler, the composition, and processing conditions.

**Figure 1:**
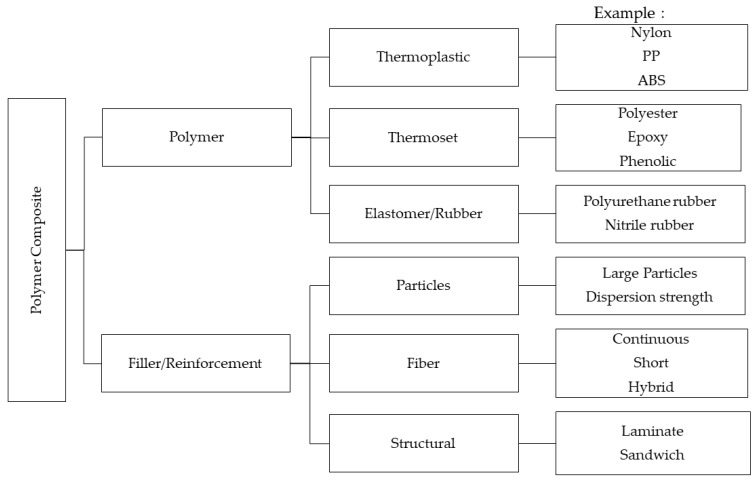
Polymer composite and types of matrix and filler.

A polymer composite is a polymeric material that is composed of at least two phases, which are the polymer as a matrix and a continuous or non-continuous filler or reinforcement [5]. As illustrated in Figure 1, the matrix material can be from various types of polymeric materials, while the reinforcement material can be from various forms of fillers. such as whiskers, particles, and fibers. The exploration of the polymer composite field arises from the polymer materials’ inability to match the performance expectations of a product. The properties of the polymer were found to be enhanced with the addition of a small amount of filler. There are many types of fillers available commercially in the market nowadays. However, mineral-based fillers are still one of the good options since they are inexpensive and abundant in nature. The fillers that are commonly used are clay, silica, talc, and calcium carbonate. Nanoclays, such as montmorillonite (MMT) and calcium carbonate (CaCO_3_), have long been used as fillers or nanofillers in polymer composite systems. Toyota’s pioneering research in 1985 introduced a polyamide/nanoclay nanocomposite for automotive application [6]. It was proved that the use of 5% MMT as a filler greatly improved the mechanical properties and thermal barrier of polyamide 6 [7]. In another work, the addition of nano calcium carbonate and nanoclay to high-density polyethylene (HDPE) significantly improved the matrix’s elastic modulus and indentation modulus [8]. As opposed to the above-mentioned mineral fillers, dolomite has been used as a filler for polymer composite systems just in the past few years [9,10,11,12,13]. Therefore, the research on dolomite as a filler in a polymer composite is still immature, requiring further investigations into how to optimize the dispersion of the dolomite in the polymer matrix, thus improving its properties. Nevertheless, several researchers have proved the capability of this naturally occurring filler in improving the mechanical and thermal properties of various polymeric materials [9,10,11,12,13]. This has attracted the interest of current researchers to develop dolomite as an alternative filler, in addition to the well-established mineral fillers such as nanoclay, talc, and calcium carbonate.

As compared to other types of minerals, dolomite possesses some advantages. It contains a large deposit of high-purity calcium magnesium carbonate and owns a unique geological structure due to the composition of two different kinds of sedimentary rocks, dolomitic and volcanic. The presence of volcanic sediment makes dolomite more resistant to weathering and endows it with hardness [14,15,16]. However, it was found that the use of dolomite in its raw form could not improve the mechanical properties of the polymeric matrices. This is because dolomite particles easily agglomerate, while their polar structure is incompatible with the non-polar structure of most polymeric materials [9,10,11,12,13,17,18]. Thus, dolomite was chemically or physically modified or treated to improve its compatibility with the polymeric matrices, and thus the performance of the resulting polymer composite [12,13,17,18,19,20]. Furthermore, several published works have reported the use of dolomite as a co-filler or hybrid filler to improve the performance of the polymer composite material [21,22,23,24,25,26,27,28,29].

This review article summarizes the use of dolomite in different fields, with a primary focus on the role of dolomite as a filler or co-filler/hybrid filler in several polymer composite systems. Several strategies used by researchers to enhance the performance of polymer/dolomite composite products are also discussed.

## 2. Filler

Fillers are materials added to resins or binders (polymer/concrete/metal/ceramic) to improve their specific properties as they are turned into a new form of material called a “composite.” Fillers are also added as binders for cost reduction. The use of fillers in various composite systems can be found widely in packaging, biomedical, cosmetic, pharmaceutical, paper, food, paint, and adhesive industries [30,31]. This is because it was recognized to improve the specific properties of the materials, such as stiffness, strength, clarity, creep resistance, and physical appearance [30,31,32]. Fillers are also being incorporated with various polymer types, such as thermoplastics, elastomers, and thermosets [31,32]. However, a thermoplastic is often used as a matrix as it can be melted at a high temperature and hardened back at a low temperature [33]. These features make thermoplastics easy to process and shape. It is worth mentioning that the filler characteristics and properties are critical aspects to know before it can be combined with the polymer to form a polymer composite material [31]. Inhomogeneous dispersion of a filler in the polymeric matrix is the main reason for the poor performance of the polymer composite. Therefore, it is essential to have excellent polymer–filler interaction and homogeneous polymer–filler dispersion to ensure outstanding quality of the produced polymer composite products [32].

Fillers can be in particulate or fibrous form. Both have their own characteristics, properties, and advantages that can be manipulated to improve the properties or performance of the polymers in order to meet specific requirements and for specific applications. For instance, the fibrous filler is frequently used to enhance the polymer’s mechanical behavior. The high aspect ratio of the fibrous filler can most efficiently improve the strength and stiffness of the polymers [34]. Glass fiber and natural fiber are examples of fibrous fillers. The particulate filler can efficiently increase the strength and toughness of the polymer matrices. Particulate fillers are typically powdered and come in a variety of shapes, sizes, and configurations. A mineral filler is one of the examples of particulate fillers [30].

The size of the filler has a great influence on the properties of the polymer composite. Typically, fillers with smaller size particles are preferred over larger ones because their surface area is larger [30,31,32]. Thus, the filler can interact with the polymer matrix phase better. In addition, researchers prefer ultrafine or nano-size fillers (nanofillers) due to their ease of dispersion and distribution throughout the polymeric matrices [33,34]. Thus, agglomeration of fillers when used at high loading can be hindered. Another aspect that needs to be considered is the compatibility between the filler and the polymer because different polarities of filler and polymer will create an inhomogeneous polymer composite with poor performance. Therefore, the filler is commonly modified (chemically or physically) in order to produce a homogeneous polymer composite. Another method to improve the performance of the polymer composite is by adding a compatibilizer that can improve the bonding between the matrix and the filler phase. The processing method is another way to ensure a well-blended polymer composite is formed [11,30,31,32,33,34].

### 2.1. Mineral-Based Filler

Mineral-based fillers are often used in polymer composites due to their abundance in nature, which can reduce the cost of polymer composite products. Other main advantages of using mineral-based fillers are their non-toxicity and capability to enhance the mechanical, physical, and thermal properties of the polymeric matrices [30,31,35,36]. As of now, kaolin, montmorillonite, calcium carbonate, magnesium hydroxide (talc), and silica are widely employed as fillers in polymer composite systems [8,30,35]. Interestingly, the addition of a small amount of mineral filler can enhance several properties of a polymer. For instance, the addition of a small amount of nanoclay (montmorillonite) was proved to provide great enhancement in the tensile strength, biostability, and thermal stability of the polyethylene-co-vinyl acetate matrix without reducing its flexibility [3,37,38,39]. In recent years, we have witnessed the use of dolomite as a mineral filler in the manufacture of composite polymers based on various types of polymer matrices. In two newly published articles, dolomite was proved to enhance the mechanical and thermal properties of the copolymer and the biopolymer-based composite [12,13]. Though mineral-based fillers are widely used in many industries, their usage should be accompanied by occupational health considerations since some fillers, such as crystalline silica, pose a health risk to workers [40,41].

### 2.2. Dolomite

Dolomite is a type of sedimentary carbonate rock that consists mainly of dolomite mineral. Dolomite was originally known as Dolomieu; it was named after a French geologist, Deodat Guy Dolomieu. Dolomite can be referred to as dolomite mineral and dolomite rock. Dolostone is another name for dolomite rock. Dolomite comes from a variety of origins. It may be present in lakes or beneath shallow seafloors, in early to late burial settings [15]. Dolomite can occur via two possible mechanisms, direct precipitation and through the dolomitization process, in which calcite dissolves, supplying Ca^2+^ ions, followed by the precipitation of dolomite from a solution rich in Mg^2+^ ions [15,16,42]. Some dolomites come from the replacement of the pre-existing limestone through the dolomitization process [15].

**Figure 2:**
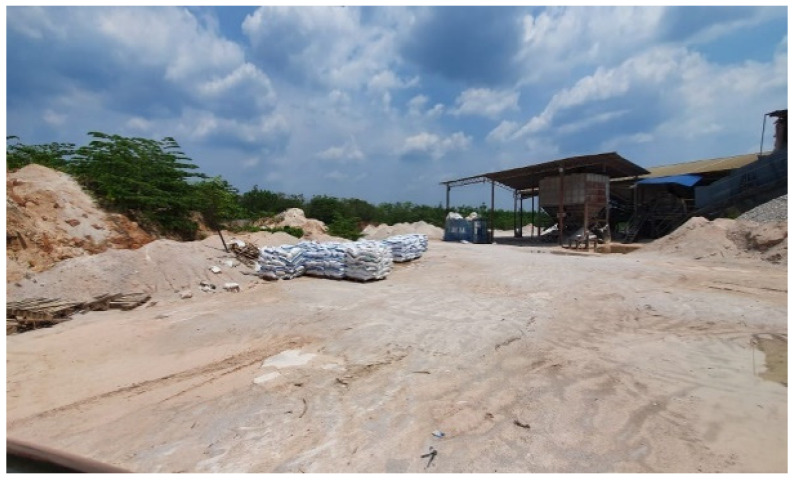
Dolomite quarry site in Perlis, Malaysia (own photograph).

Dolomite is one of the most abundant carbonate rocks after calcite. Dolomite is widely spread in nature and can be found in Nigeria, Canada, Malaysia, and many other countries [10,43,44]. In Malaysia, dolomite can be found abundantly in the states of Perlis and Perak. There are several large quarries, thus placing them as a major producer of Malaysian dolomite [11,45,46]. Figure 2 shows one of dolomite’s quarry sites in Perlis. Dolomite that is found in Perlis is also known as “Batu reput” by the locals and is a source of quality mineral rocks [42].

**Figure 3:**
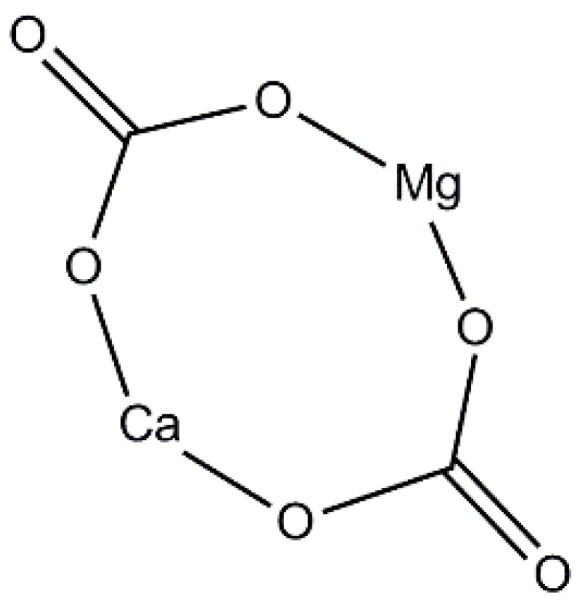
Structure of dolomite.

**Figure 4:**
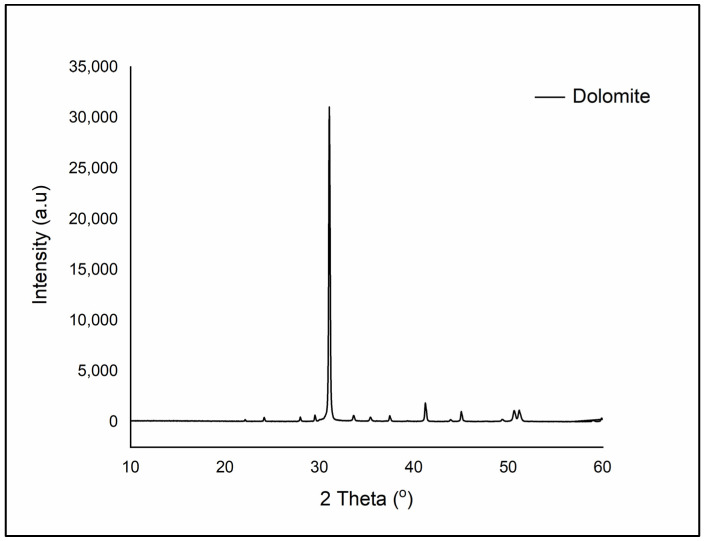
XRD diffraction graph of raw dolomite.

Dolomite is known to be a derivative of calcite. Therefore, dolomite mineral has a similar chemical composition to calcite mineral. Calcite minerals consist of calcium and carbonate (CaCO_3_) layers only, while dolomite mineral consists of three layers, made up of alternating calcium (Ca) and magnesium (Mg) layers separated by a carbonate (CO_3_) layer. Thus, the structure of dolomite is not exactly in ordered form as in calcite, because some of the magnesium may be present in the calcium layer, while some of the calcium may be present in the magnesium layer [27]. The chemical structure of dolomite is presented in Figure 3. Hydrochloric acid is used to distinguish between calcite and dolomite. Adding HCl to calcite will cause a fuzzy reaction, while adding it to dolomite will only cause a bubbly reaction. Figure 4 shows the XRD diffraction peak of raw dolomite that was obtained from Perlis Dolomite Industries Sdn. Bhd., Malaysia. There is a sharp peak observed at 31.04°, which indicates the presence of dolomite mineral. This is in good agreement with the findings of Gregg et al. and Abdul Samad and Abd Rashid [47,48]. Dolomite from the Perlis Dolomite Industries also contains 41.4% CaCO_3_, which is said to be of great quality [49].

**Figure 5:**
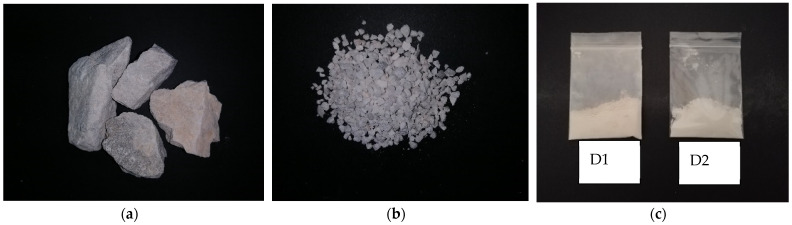
(**a**) Dolomite in gravel form, (**b**) dolomite in smaller granule form, and (**c**) dolomite in fine powder form.

**Figure 6:**
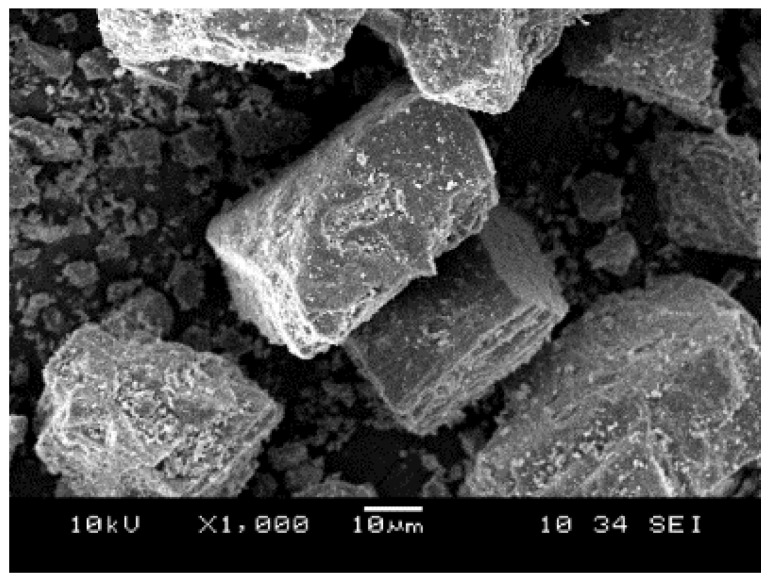
SEM image of raw dolomite particles.

**Figure 7:**
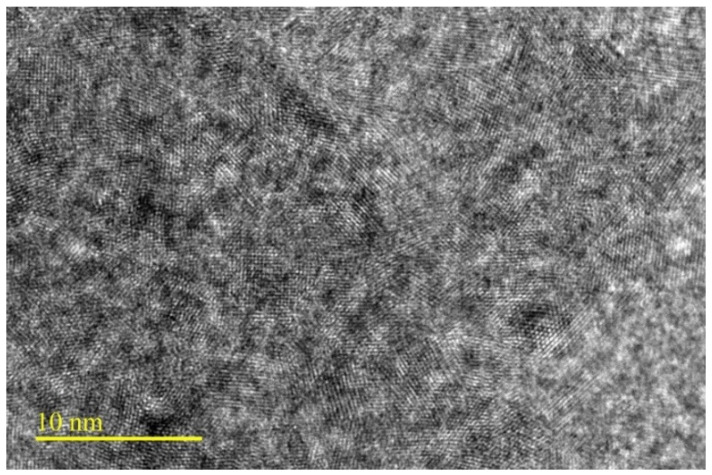
TEM image of raw dolomite particles.

Dolomite in the form of gravel stone may have different colors, for example, white, colorless, pink, gray, and brown, but when it is transformed into fine powder, its color turns to yellowish. We have collected some dolomite samples from Perlis Dolomite Industries Sdn. Bhd. and performed SEM and TEM analyses. Figure 5 shows the different particle sizes of dolomite. Figure 5a,b present dolomite in the form of gravel and granules, respectively. Figure 5c shows the powder form of dolomite. D1 represents dolomite with a particle size of 150 µm, while D2 represents dolomite with an average particle size of 63 µm. D1 has rougher texture, while D2 has smoother and soft texture. Table 1 summarizes a comparison of the physical properties of dolomite and calcite. Dolomite has a rhombohedral cleavage and a hexagonal crystal system, while calcite has a rhombohedral and trigonal system. It has Mohs hardness of 3.5–4 and specific gravity of 2.8–2.9. Figure 6 displays the SEM images of dolomite, suggesting the smooth surface of this mineral’s particles. Furthermore, the morphology of the dolomite indicates that it has an irregular shape and a rhombohedral structure. The TEM image in Figure 7 suggests that raw dolomite’s particle morphology possesses a Moire fringe pattern. This is due to the overlap of two different phases in the structure of dolomite, which are Mg and Ca [50].

**Figure 8:**
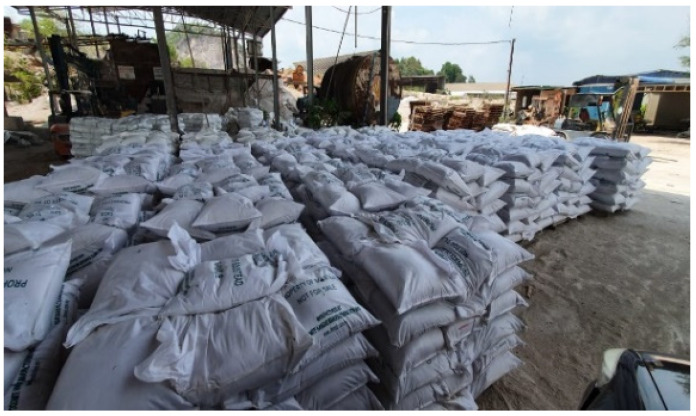
Packed dolomite for use as fertilizer (own photograph).

Dolomite was extensively used in many fields and industries many years ago, in the form of either raw dolomite or calcined dolomite. For example, in agriculture, dolomite was used in the form of raw material for soil stabilization and fertilizer [53]. Figure 8 shows the image of packed dolomite ready to be used as fertilizer. Dolomite was also used for decreasing the acidity of soil and the adjustment in magnesium concentration in soil. In addition, dolomite that was used for construction was also in raw dolomite form. Dolomite also has the ability to absorb poisonous and toxic substances [8,54,55]. Table 2 summarizes the use of dolomite in different industries.

## 3. Dolomite as a Filler in a Polymer Composite

Recent studies have proved that the use of dolomite as a filler in the polymer composite field is so promising because this naturally occurring material can allow improvement in several polymer properties [9,10,11,12,13]. The performance of polymers was seen to be greatly influenced by the addition of dolomite. There are several types of testing/analysis used to assess the polymer/dolomite composites, such as mechanical analysis through tensile, impact, flexural, compression, and hardness tests. The physical performance of the polymer/dolomite composites can be tested by performing the thermal stability and flammability tests. Generally, the performance of a polymer/dolomite composite is influenced by several factors, for example, the size of the dolomite used, the loading of dolomite, the polymer used, the interaction between polymer and dolomite, the compatibility between dolomite and polymer, and the processing method [11,13,17]. Other than being abundant in nature, the high rigidity and hardness of dolomite make it an ideal filler for reinforcing low-modulus polymers [12,13,17,44,64]. Another interesting fact is that the inorganic nature of dolomite is useful for improving the thermal stability of organic polymers [62]. However, as opposed to other well-recognized mineral fillers, such as nanoclay and calcium carbonate, the research on dolomite as a filler in polymer composite materials is still immature. As mentioned earlier, the use of dolomite has been more concentrated for construction and agricultural applications over the decades. Only in recent years has it been used as a filler for polymer composites. Thus, the surface chemistry of dolomite and its interactions with synthetic organic polymers and surfactants have not been well studied and documented, unlike those of nanoclays or layered silicate fillers. Nevertheless, there are some research and publications related to dolomite as a filler of polymer composites that can provide good and useful information toward the development of this polymer-/mineral-based composite for future use in various applications. This section will discuss the role of dolomite as a filler and its effect on the performance of polymer composites.

### 3.1. Pristine (Unmodified) Dolomite as a Filler in a Polymer Composite

There are several reports on the use of pristine dolomite as fillers in polymer composite systems [10,64,65]. The research related to this field is summarized in Table 3. Adesakin et al. have evaluated the mechanical properties of the polyester composite with dolomite as a filler [10]. The composites were prepared with 5%, 10%, 20%, 30%, 40%, and 50% of dolomite loading. The polyester/dolomite composite showed a reduction in tensile strength when the dolomite content increased. At 40% dolomite loading, the tensile strength was at the lowest value, where it decreased by 77.6% when compared to that of the virgin polyester. On the contrary, the modulus of the polyester increased as dolomite was added to the polyester. At 30% dolomite, the increment was 44.3% when compared to that of the virgin polyester. Their hardness was also found to be increased by 211.3% and 50% when the dolomite was loaded at 20% and 30%, respectively. The hardness of dolomite contributes to the increment in the hardness of the polyester composite.

Ali et al. prepared and characterized a polyether-based polyurethane/dolomite composite [65]. The composite was prepared by the casting process with five different dolomite loading values (0%, 10%, 20%, 30%, 40%, and 45%). They also obtained a similar trend in tensile test results, in which the tensile strength further decreased on increasing the dolomite loading. The virgin-polyether-based polyurethane has a tensile strength value of 30 MPa, but when dolomite was added at 45%, the tensile strength value reduced to 16 MPa (−46.7%). Their elongation at break also followed the trend of tensile strength. The value decreased by 76.5% when compared to that of the virgin-polyether-based polyurethane. However, their compression strength was reported to increase with increasing dolomite loading. The value increased from 7 kg/m^2^ (0%) to 14.27 kg/m^2^ (45%). This shows that the composite has better compression stability at lower dolomite loading.

Mohd Din et al. investigated the mechanical properties, thermal properties, and flammability of polypropylene (PP)/dolomite composites [64]. The composites were prepared by using a twin-screw extruder and injection molding with four different dolomite loading values (0%, 5%, 10%, and 15%). They also obtained similar findings, in which the dolomite did not successfully improve the tensile strength of the host polymers. The tensile strength value reduced from 19 MPa to 16.2 MPa when the dolomite loading was 15%.

The overall tensile strength of the polymer matrix, as reported by Adesakin et al., Ali et al., and Mohd Din et al., decreased with increasing dolomite loading. This is mainly due to poor interfacial adhesion between dolomite filler and polymer. It is known that polyester, polyether-based polyurethane, and polypropylene are hydrophobic polymers and, therefore, the incompatible with dolomite, which is hydrophilic in nature. This creates a weak filler–matrix interaction. These weak filler–matrix interactions lead to a deterioration in the matrix’s mechanical properties. Research that has used other types of filler has also found similar results, in which the use of a mineral filler in its pristine form did not improve the mechanical performance of the polymer composite [66,67]. In addition, raw dolomite used as a filler usually contains large particle sizes (micron to mm range), thus leading to poor dispersion of dolomite in the matrix phase. Consequently, the agglomerated particles hinder the mobility of the polymer chains when load is applied to the polymer composite. Through the morphological study of the fractured surface of the polypropylene/dolomite composite, the voids can be seen throughout the matrix, which shows that there is no adhesion between dolomite and polymer matrix [64]. The flexural modulus of polypropylene increased when the dolomite was loaded at 5% and 10%, in which 10% was the optimum loading. However, when the dolomite content increased to 15%, the flexural modulus decreased due to the agglomeration that commonly occurs at higher filler loading [64]. This agglomeration also caused the presence of voids in the matrix phase. However, the flexural strength of the polypropylene composite was reported to be decreased due to the poor adhesion between dolomite and polypropylene.

Research by Mohd Din et al. also found that the thermal stability and flame retardancy properties of the polypropylene were improved with the addition of a dolomite filler [64]. This shows that dolomite helps to increase the flame-retardant properties by taking a longer period for burning both polymers completely. The degree of crystallinity of each polymer was reported to be increased as dolomite was added. This shows that dolomite acted as a nucleating agent for PP. As the degree of crystallinity increased, the hardness of the composite was also improved.

Generally, the performance of a polymer composite with pristine dolomite does not really improve as the mechanical properties show no or little improvement. This is mainly due to the agglomeration of dolomite in a polymer composite system and poor interfacial adhesion between dolomite and polymer. These can be improved by physical and chemical modification of dolomite. Previously, modified fillers such as nanoclay have shown better interaction with polymers when compared to the use of non-modified fillers [38]. Silane modification of a talc filler also helps to improve the interaction between matrix and filler [68]. Thus, the polymer performance is enhanced.

Based on these three research works, we can see that another aspect needs to be considered when using pristine (unmodified) dolomite as a filler, i.e., the type of polymer matrix (either thermoplastic or thermoset), for producing the polymer/dolomite composite. Apparently, at the same dolomite loading (e.g., 10%), the use of a thermosetting matrix (polyester) has resulted in a more significant reduction in the tensile strength of the composite as compared to the use of a thermoplastic matrix (polypropylene). Generally, the nature of both thermoset and thermoplastic and their processing method can determine how well the dolomite can be dispersed throughout the matrix. The use of a twin-screw extruder for processing a thermoplastic/dolomite composite can provide a greater shear force to de-agglomerate the dolomite, thus assisting in its dispersion in the matrix. In contrast, the casting process or manual stirring used to process the thermoset/dolomite composite cannot provide enough shear to allow breaking of the highly agglomerated particles of dolomite. These poorly dispersed agglomerated particles will reduce the efficiency of the stress transferring mechanism between the matrix and the filler, leading to inferior mechanical properties of the polymer.

### 3.2. Physically Modified Dolomite as a Filler in a Polymer Composite

The use of pristine dolomite was proved to not significantly improve the performance of polymer composites, particularly mechanical performance. Therefore, there are researchers who used a physical approach to modify the physical aspect of dolomite, such as reducing its particle size and agglomeration through the application of mechanical energy (milling), ultrasonic force, and shear energy [9,11,12,69,70,71]. This modification was proved to assist in the better dispersion of dolomite in the polymer composite, promoting better interaction and adhesion of dolomite and polymer. Furthermore, there are several researchers who have studied and compared the mechanical performance of polymer composites with different sizes of dolomite [11,12,71]. Table 4 summarizes the type, processing method, and properties of polymer composites with dolomite fillers that have been physically modified.

Ridhwan et al. studied the effect of dolomite filler size on the properties of standard Malaysian rubber (SMR L) and epoxidized natural rubber (ENR 50) composite [11]. The composite samples were prepared using two-roll mill at room temperature and followed by compression molding at 160 °C. Different dolomite loadings were employed, which were 5 phr, 15 phr, 25 phr, 35 phr, and 50 phr. As mentioned in their articles, the dolomite’s particle size used was in the range of less than 63 µm (S1) for smaller size dolomite and 75 to 150 µm (S2) for larger size dolomite. They studied the tensile properties of the composites and found out that the addition of dolomite with two different sizes to both types of rubber improves their tensile properties by up to 15 phr. This is because those dolomite particles can be well distributed inside the polymer matrices if added in low amounts. Overall, the dolomite/SMR L composite has higher tensile performance when compared to the dolomite/ENR 50 composite. The dispersion of dolomite particles was greater in the SMR L than in the ENR 50 rubber, thus resulting in a better filler/matrix interaction. In addition, both SMR L and ENR 50 with smaller particles of dolomite (<63 µm) displayed a better tensile performance when compared to the ones containing bigger particles of dolomite (75–150 µm). The reason behind this was that smaller fillers have a higher surface area, which allows for better filler–matrix interaction. Smaller size fillers can also be uniformly distributed and dispersed in the matrix. This is in agreement with the results of other research studies involving polymer composites incorporating other types of mineral fillers, such as bentonite and fumed silica [72,73].

Research by Syed Bakar et al. highlights the use of dolomite as a filler in a recycled polypropylene matrix (rPP). The composites were prepared via a Z-blade mixer with dolomite loading of 10%, 20%, 30%, 40%, and 50% [71]. The dolomite was sieved into two different sizes, which were 63 µm and 300 µm. The overall tensile performance of the rPP with both 63 µm and 300 µm dolomites improved when compared to that of the virgin rPP. The tensile strength of the rPP composite was at the highest value when the dolomite loading was at 30%. However, the rPP composite with a smaller particle size (63 µm) showed a lower tensile strength value when compared to that of the rPP with a larger dolomite size (300 µm). Smaller size particles tend to agglomerate and form bigger size particles at higher loading [74]. These phenomena lead to a decrement in the tensile performance of rPP with smaller size dolomite. In the research of both Ridhwan et al. and Syed Bakar et al., the tensile strength of the polymer composite was reported to decrease at dolomite loading of 25 phr and 40% [11,71]. The value was further decreased as dolomite loading increased. This is because at higher dolomite loading, agglomeration of dolomite particles occurs and this is shown in the SEM images of the composite’s fractured surface at 50% dolomite loading [71]. Meanwhile, the SEM images of both SMR L and ENR-50 composites with dolomite show a rougher and courser surface, which indicates the occurrence of stiffness and brittle failure [11].

A research article by Ahmad Fauzi et al. highlights the effect of ultrasonicated dolomite on the performance of polyethylene-co-vinyl acetate (PEVA) [69]. The particle size of raw dolomite obtained from the supplier was around 150 µm. The raw dolomite was ultrasonicated using 30% amplitude over three lengths of time, which were 2, 3, and 5 h. Dolomite that was ultrasonicated for 2 h possessed the smallest particle size, which was around 50 µm. Thus, it was chosen to be the filler for the PEVA composite. The mechanical performance of the composites was assessed through a tensile test. Results indicated that the tensile strength, modulus of elasticity, and tensile toughness of the PEVA/dolomite composite showed an increasing trend when the dolomite content increased from 1 to 5 wt%. The reason behind this was that the ultrasonication process caused de-agglomeration of dolomite; therefore, the dispersion of dolomite in the PEVA was improved. This consequently led to the improvement in the tensile properties of the PEVA copolymer matrix. The hard and stiff particles of dolomite also restricted the chain mobility of the PEVA matrix, thus causing an increment in the modulus elasticity [11]. The smaller size dolomite particles are more mobile and easily dispersed in the PEVA matrix. Thus, the filler assisted in the energy absorption mechanism during tensile deformation and enhanced the toughness of the PEVA matrix. However, the elongation at break of the PEVA composite showed a decreasing trend with increased dolomite loading. The addition of a stiff filler to the soft elastomeric PEVA caused the ductility of the PEVA composite to decrease, thus decreasing the elongation at break [69].

In another study, Ahmad Fauzi et al. analyzed the tensile performance of PEVA with ground and ultrasonicated dolomite (GUD) as a filler [70]. The dolomite was ground by using ball milling and ultrasonicated for 2 h at 30% amplitude. The dolomite size obtained was around 20 µm. The composites were compounded with heated two-roll mill with different dolomite loading values (1%, 3%, and 5%). The tensile strength, the elongation at break, the tensile toughness, and the modulus of elasticity were reported to be increased when dolomite was added to the PEVA. The optimum tensile properties were achieved when 1% dolomite was employed as a filler. As proved through their previous research, dolomite with a smaller particle size (GUD) can be easily dispersed and distributed in the PEVA composite system, especially at lower loading. As GUD was well dispersed and distributed, a homogeneous PEVA composite formed and led to the increment in the elongation at break, especially at 1% dolomite loading. However, the overall tensile performance reduced as dolomite loading reached 3% and further decreased when the dolomite was further loaded to 5%. Agglomeration of dolomite particles might occur at higher dolomite loading, which causes poor dispersion and distribution of dolomite in the PEVA matrix. Furthermore, the processing method might as well influence the performance of the PEVA composite. Ahmad Fauzi et al. used two different processing methods, which were heated two-roll mill and twin-screw extruder. By using the heated two-roll mill, the best enhancement in the tensile strength could be achieved when the dolomite loading was 1 wt%. However, when using the twin-screw extruder, 5 wt% of dolomite resulted in the best increment. According to Vergness et al., the twin-screw extruder can provide a better mixing of polymer/filler as it has a mixing zone, which is called the kneading disc [75]. It was reported that a high number of kneading discs provide a better exfoliation of the filler in the matrix phase due to the greater shearing effect [76,77]. However, less shear force was imparted when using the heated two-roll mill due to the flat surface of the roller. Thus, the filler might be well dispersed but not well distributed throughout the matrix.

Ahmad Saidi et al. examined the flammability, thermal, and mechanical properties of polybutylene terephthalate (PBT) composites with dolomite as a filler [9]. In this research, dolomite was ground into 200 µm from the rock form before being employed as a filler. The composites were prepared with 0%, 5%, 10%, and 15% dolomite loading via a twin-screw extruder. The tensile strength of composites was reported to increase on up to 10% dolomite loading and start to decrease at 15% dolomite loading. On the contrary, the impact strength of the PBT decreased with increasing of dolomite loading. This was due to the presence of dolomite, which initiated crack propagation in the PBT matrix as dolomite acted as stress concentrators [11]. The thermal properties and flammability were reported to be improved as well.

Another fact that is worth highlighting is that dolomite can be used to reinforce both synthetic and natural polymers/biopolymers. The work by Osman et al. demonstrates that dolomite can reinforce the biopolymer matrix when the tip-sonication method is employed to de-agglomerate the dolomite prior to mixing with the biopolymer. In this study, dolomite at 1–5 wt% loading was used as a filler in the thermoplastic starch matrix (TPS). There are two types of dolomites being used as a filler: first, raw dolomite (P) and second, dolomite that was ground, sieved, and tip-sonicated (U). The composites with high loading of dolomite of both sizes (4 and 5 wt%) possess greater tensile and tear properties as compared to the composites with low loading of dolomite (1 and 2 wt%). Furthermore, it is proved that a composite with tip-sonicated dolomite can achieve greater mechanical properties than a composite with raw dolomite. De-agglomeration of the dolomite upon the sonication process reduced its particle size, thus assisting in its dispersion and distribution throughout the TPS matrix. This led to improvement in the tensile and tear properties of the composite [12].

**Figure 9:**
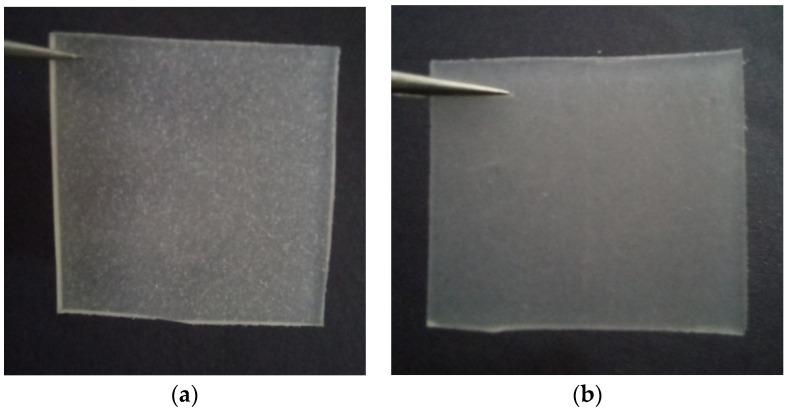
(**a**) Polyethylene-co-vinyl acetate (PEVA) composite with pulverized dolomite (particle size = 150 µm). (**b**) PEVA composite with ground dolomite (particle size ≤ 63 µm) (own photograph through own research).

**Figure 10:**
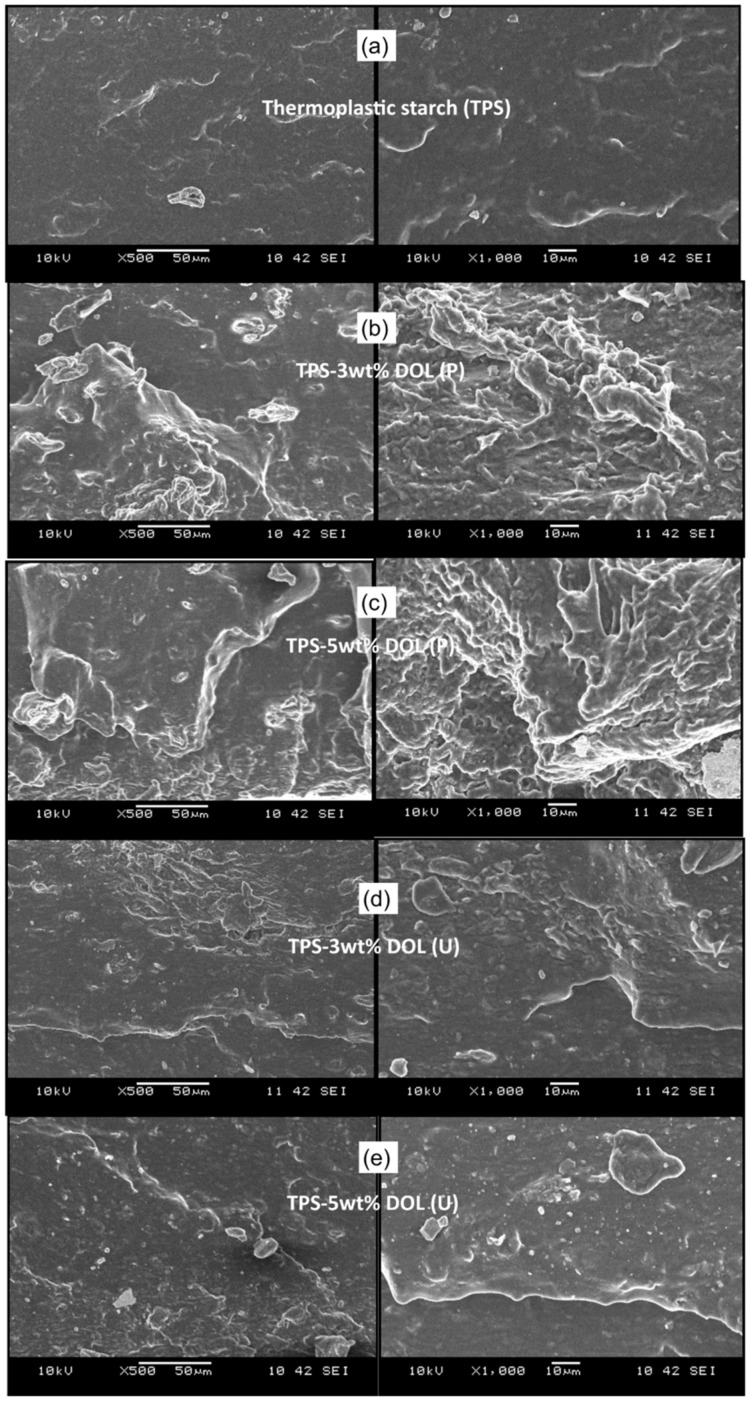
The SEM images of the tensile fractured surface of the (**a**) TPS film, (**b**) TPS-3 wt% DOL (P), (**c**) TPS-3 wt% DOL (U), (**d**) TPS-5 wt% DOL (P), and (**e**) TPS-5 wt% DOL (U) biocomposite films at 500× magnification (**left**) and 1000× magnification (**right**). Reprinted with permission from Ref. [12].

Based on the literature studies summarized in Table 4, we can conclude that the use of dolomite with smaller particle sizes can more efficiently improve the mechanical properties of the polymers. Generally, the use of bigger size fillers causes deterioration in the mechanical performance of the polymer matrix. This is due to the fact that larger particles have smaller surface areas and, thus, lesser interphase bonding/interaction with the matrix phase. In addition, larger size fillers may hinder the mobility of the polymer chains when the composite is being deformed or stretched. This will initiate crack propagation. However, smaller size fillers will have a higher specific surface area and surface/volume ratio. This will provide a better matrix–filler interaction. In addition, smaller particles can be dispersed and distributed better in the matrix due to greater mobility [78]. Another reason for the deterioration in the polymer composite performance is the incompatibility between the matrix and the filler phase, which creates a phase-separated polymer composite called a microcomposite. This type of composite morphology can be more clearly seen if the particle size of the dolomite used is large. Figure 9a shows an example of a dolomite/polymer microcomposite system that we have prepared in our lab, where large particles of dolomite can be seen distributed in the polyethylene-co-vinyl acetate (PEVA) matrix. In this case, the dolomite particle size was 150 µm. On the contrary, a better dispersed dolomite filler in the PEVA matrix can be seen in Figure 9b. In this case, the dolomite particle size was <63 µm. These results indicate that dolomite with smaller particles can be more easily dispersed in the polymeric matrix and can create a homogenous PEVA composite. The work by Osman et al. supports our findings [12]. Figure 10 presents the SEM images of the fractured surface morphology of the TPS/dolomite composite. Figure 10b,c indicates that the incorporation of bigger particles of dolomite causes a rougher surface fracture of the matrix, which is due to the poor dispersion of the filler in the matrix. Figure 10d,e shows that the incorporation of smaller particles of dolomite causes a smoother surface fracture, which is due to the good dispersion and distribution of the filler in the matrix [12].

Though improvement in the polymer’s properties is noticeable with the addition of physically modified dolomite, their composition in the polymer matrix should be controlled. Research findings indicate that the polymer composite will show optimum performance when the dolomite loading is below 30 wt%. This is due to the agglomeration of dolomite particles in the matrix phase when the filler is added at high loading [13,69,70]. For instance, Syed Bakar et al. have observed clear agglomeration of dolomite particles in the SEM morphology image of the tensile fractured surface of their composite sample with 50 wt% dolomite [71]. This agglomeration of dolomite particles is caused by filler–filler interactions rather than filler–matrix interactions. As mentioned earlier, dolomite is naturally hydrophilic and, thus, it is incompatible with most polymers. Thus, the interaction between polymer and dolomite is weak. This weak filler/matrix bonding will prevent efficient stress transfer mechanism when the tensile load is applied on the composite sample. Furthermore, agglomerated particles hinder the molecular chain mobility of the host polymer, thus preventing the occurrence of a smooth energy absorption mechanism through molecular motions. As a result, the composite can easily break when stretched, showing low elongation-at-break and tensile toughness values [13]. Thus, a chemical modification of the filler is commonly performed to improve the interaction between the filler and the matrix.

### 3.3. Chemically Modified Dolomite as a Filler in a Polymer Composite

Chemical treatment or surface modification has been widely applied to enhance the interfacial adhesion between the filler and the polymer matrix. The commonly used methods to modify fillers through the chemical approach are via surface modification, chemical grafting, coupling agent, or compatibilizer addition [12,37,38,79]. All of these modification methods have been proved to improve the dispersion of the filler in the polymeric matrices, thus enhancing the homogeneity of the resultant polymer composite materials. Therefore, researchers who have employed dolomite fillers have also used the same approach/method to produce the homogeneous polymer/dolomite composites. Table 5 summarizes the different methods used to modify dolomite and how each method improves the performance of the polymer/dolomite composite.

Manni et al. studied the physical properties of the polyamide 11 (PA11) composite incorporating a silane-modified dolomite filler. In this research, the dolomite samples underwent modification with silane first, before being compounded with polyamide 11. The composite samples were prepared by using a micro-extruder with dolomite loading of 5%, 10%, and 15%. According to Manni et al., the thermal stability of polyamide 11 was successfully improved with the addition of the silane-treated dolomite filler [18]. This was due to the excellent dispersion and distribution of the dolomite filler in this polymer as the silane compound assisted in the bonding of both inorganic and organic functionalities within the same molecules [18,79,80]. The use of silane also increases the adhesion between particle and polymer matrix [80]. The SEM images of the composite indicate a good interfacial adhesion between the dolomite and the polymer, and the dolomite is seen to be well dispersed and distributed in the matrix [18].

Nik Adik et al. conducted research to study the effect of stearic-acid-modified dolomite on the tensile, morphological, and thermal properties of the polypropylene (PP) composite. The composites were prepared by using brabender with dolomite loading between 5% and 25%. The dolomite was first ground to 63 µm before being surface modified with the acid. In a more recent work, Lim et al. also used stearic acid as a surface modifier for dolomite. This research examined the mechanical and thermal properties of poly(ethylene-co-vinyl acetate) (PECoVA) composites. In this study, the dolomite was ground and ultrasonicated until the dolomite reached the size of ~15 µm. Then, it was chemically modified with stearic acid. The composites were prepared by using heated two-roll mill with different dolomite loading values (1%, 3%, and 5%). Research by Nik Adik et al. and Lim et al. concluded that the use of stearic acid to treat dolomite improves the properties of the polymer matrix mechanically and thermally [13,17]. However, there is a contradictory finding between these two studies of Lim et al. and Nik Adik et al. The tensile strength of the PECoVA composite with the stearic-acid-modified dolomite was found to be higher than that of the PECoVA composite with raw dolomite [13], but the tensile strength of the PP composite declined when the stearic-acid-modified dolomite was employed as a filler. Generally, as modified dolomite is added to the polymer, the tensile performance is expected to be improved, but the research by Nik Adik et al. showed a contradictory result and it might be due to the insufficient amount of stearic acid used to modify the dolomite. Stearic acid performs better at concentrations of less than 2% [17].

**Figure 11:**
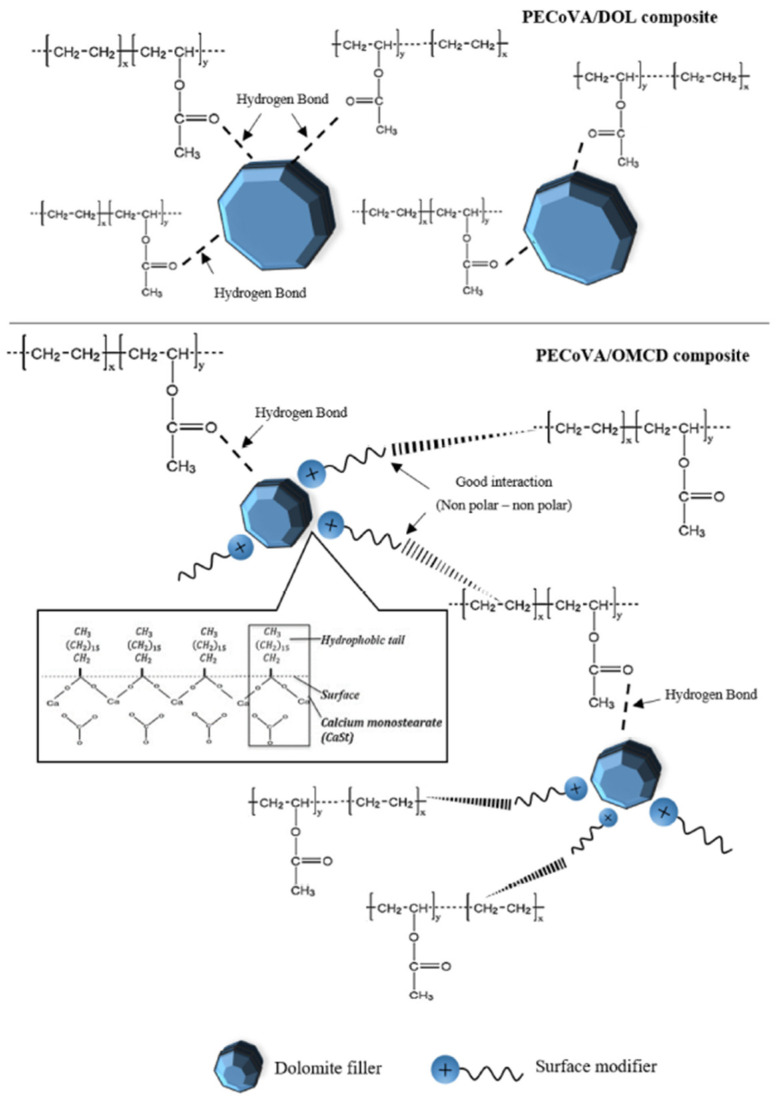
The proposed interactions between the PECoVA copolymer chain with DOL and OMCD fillers in the PECoVA composite system. Reprinted with permission from Ref. [13].

The homogeneity of the PECoVA composite with the stearic-acid-modified dolomite was also reported to be better than the one containing pristine dolomite. The reason for this observation is that stearic acid has rendered the polar surface of dolomite into non-polar due to the introduction of a new functional group on the surface of the dolomite. Figure 11 shows the illustration of the proposed mechanism for the interaction between PECoVA and stearic-acid-modified dolomite [13]. It shows that there is only hydrogen bonding between dolomite and polymer when pristine dolomite is used as a filler whereas there is non-polar interaction and hydrogen bonding when modified dolomite is used as a filler. These two interactions improve the interfacial adhesion between dolomite and polymer. The hydrophobic characteristic of the modified dolomite makes it more compatible with hydrophobic polymers such as PP and PECoVA, where good interactions between the filler and the matrix can occur. As a result, the stearic-acid-modified dolomite can be better dispersed and distributed in the matrix of both polymers, thus being able to serve as a reinforcing filler. The fractured surface of the PECoVA copolymer was also revealed to be smoother after being subjected to tensile forces. Young’s modulus of the PECoVA composite also increased when the stearic-acid-modified dolomite was added up to 3 wt%. The increment was due to the restricted mobility chain by the addition of the modified dolomite, and the stiffness of the dolomite also contributed to the increment of the stiffness of the copolymer [69]. However, for a PP composite, Young’s modulus decreases when modified dolomite is employed as a filler. As previously discussed, this might be due to the insufficient amount of stearic acid used.

**Figure 12:**
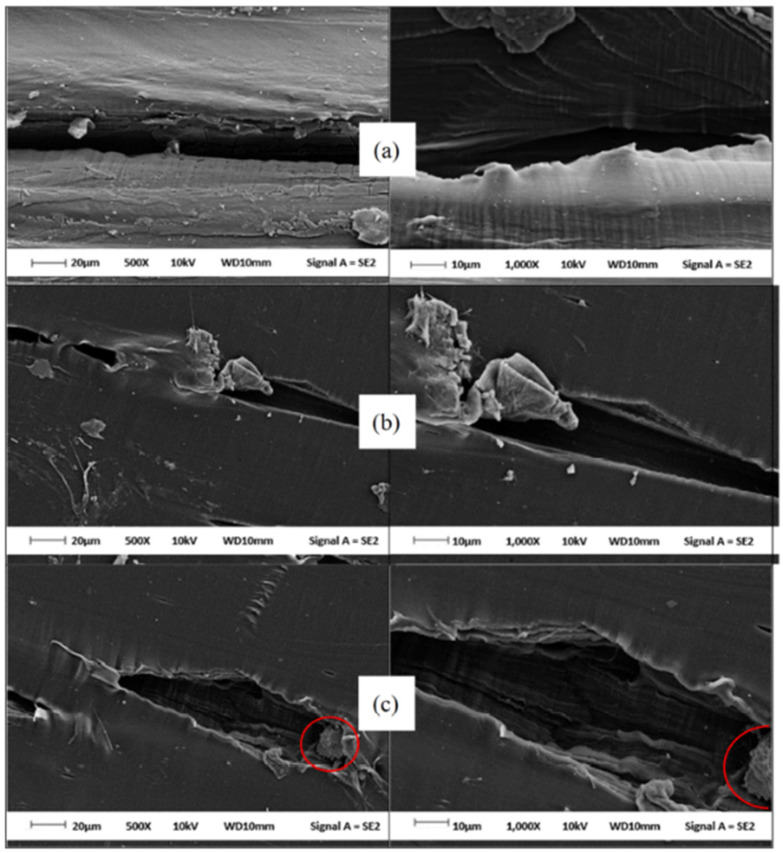
The FESEM images of the tensile fracture surface of the (**a**) virgin PECoVA, (**b**) PECoVA/DOL 3, and (**c**) PECoVA/OMCD 3 composite at 500× magnification (**left**) and 1000× magnification (**right**). Reprinted with permission from Ref. [13].

**Figure 13:**
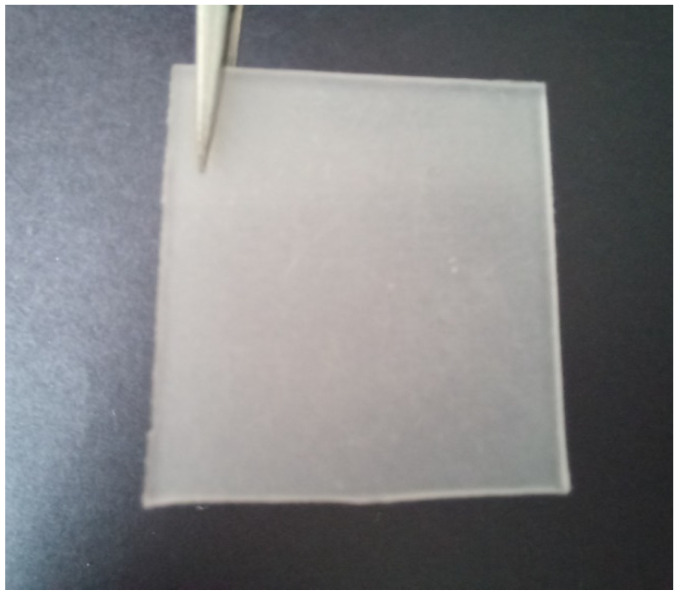
PEVA composite film with a stearic-acid-modified dolomite filler.

Figure 12 reveals the FESEM images of the virgin PECoVA, PECoVA/DOL 3, and PECoVA/OMCD 3. Figure 12c demonstrates that PECoVA with stearic-acid-modified dolomite (PECoVA/OMCD) possesses a smoother and more homogeneous surface after being fractured by tensile forces as compared to the virgin PECoVA (Figure 12a) and the PECoVA composite with pristine dolomite (PECoVA/DOL) (Figure 12b). As mentioned earlier, the use of modified dolomite improves the interfacial adhesion between dolomite and polymer and it will lead to a uniform stress distribution in the matrix. This homogeneous dispersion and distribution of modified dolomite in a polymer also leads to improvement in the thermal stability of the polymer composite. Figure 13 shows a polyethylene vinyl acetate (PEVA)/dolomite composite film containing stearic-acid-modified dolomite as a filler (obtained from our own research). Good dispersion of dolomite makes the composite appear more homogeneous as opposed to the composite films containing unmodified dolomite (see Figure 9).

PP and PECoVA incorporated with modified dolomite also possess better thermal properties than those incorporated with raw dolomite. The stearic acid treatment modifies the surface of dolomite, where a layer of hydrophobic organic molecules attaches to the dolomite’s surface, changing the characteristic of dolomite from hydrophobic to organophilic [13,17]. This modification of dolomite makes it more compatible with both PP and PECoVA. Good dispersion of the inorganic filler inside the organic matrix can slow down the thermal decomposition of the polymeric molecules. This is the reason why the thermal stability of PP and PECoVA can be improved with the addition of modified dolomite. In addition, Lim et al. analyzed the dynamic thermomechanical behavior of the PECoVA/dolomite composite. Due to the good compatibility between the matrix and the filler, the modified dolomite particles were found to integrate well in the molecular chains of the PECoVA. Therefore, when subjected to dynamic loading under an elevated temperature, the viscoelastic damping factor of the matrix phase reduced. This is because the modified dolomite filler has restricted the molecular mobility of the PECoVA chains [13].

Ankabi et al. investigated the PP/dolomite composite, to which maleic anhydrate was added as a compatibilizer [19]. The dolomite filler was incorporated with polypropylene at filler loading between 0% to 20%. Due to the addition of the compatibilizer, the tensile strength, flexural strength, and hardness of the matrix increased with increasing of dolomite loading. The values of tensile strength, flexural strength, and hardness of the PP polymer composite increased by 37%, 40%, and 40.02%, respectively. However, the elongation at break of the PP composite with dolomite (20%) and compatibilizer decreased by 50% when compared with that of virgin PP. This was due to the reduction in the deformation of a rigid interface between the fillers and the matrix. This proved that the compatibilizer enhances the interfacial adhesion between the PP and the dolomite filler and leads to the improvement in the mechanical performance of the host polymer.

Another work by Ghada et al. evaluated the effect of dolomite on the mechanical properties and the thermal stability of polyvinyl chloride (PVC). The composites were prepared at a rigid blend composition varying from 0 to 20 phr via an internal mixer. They indicated that significant enhancement in the mechanical properties and thermal stability of the PVC can be achieved with the addition of a dolomite filler and several additives [20]. In their work, the addition of 5 phr dolomite in combination with other additives (thermal stabilizer, co-stabilizer, anti-oxidant, anti-UV, and internal and external lubricants) resulted in the increment in tensile strength, elastic modulus, impact strength, elongation at break, and thermal stability when compared to those of virgin polymer [20]. However, the tensile strength and elongation at break showed a decreasing trend when dolomite was added at higher loadings (≥10 phr). Yet the values were still greater than that of virgin PVC. The agglomeration and overcrowding of dolomite filler in the matrix phase was the cause of this phenomenon.

Based on the literature studies summarized in Table 5, all the chemical modification using acid functionalization and compatibilizer involved the polymer/dolomite composite with a thermoplastic matrix. There is no published research found on the chemically modified dolomite for a thermoset-based composite. As summarized in Table 4, most of the research related to the thermoset/dolomite composite used a physical approach to modify the dolomite, such as through particle size reduction. The need for other chemicals, such as hardeners and crosslinkers, in the curing process of the thermoset may restrict the use of other chemicals for the treatment of a dolomite filler. This is because unwanted chemical reactions during processing must be avoided.

Other than the types of treatment/modification mentioned above, there is another alternative to improving the performance of polymer/dolomite composite materials. A study by Sönmez et al. (2020) revealed that dolomites that have been surface modified with titanium and silicon precursors possess great potential as photocatalyst fillers in the poly(dimethylsiloxane) matrix for water treatment application. The functionalization of dolomite with titania and silica molecules has improved not only its photo-catalysis property but also its mechanical properties [81].

The research studies explained above indicate that there are several modification approaches that can be used to increase the potential and function of dolomite as a filler in a polymer composite system. However, the literature suggests that there is another option to upgrade the potential of dolomite as a filler in a polymer composite/nanocomposite system. Dolomite is prepared as a co-filler or a hybrid filler with another type of filler/nanofiller to provide synergistic effects to the resultant composite/nanocomposite material. The following subsection discusses this topic.

### 3.4. Dolomite as a Hybrid Filler in a Polymer Composite

Over the years, the use of more than one filler or hybrid filler in a polymer composite system has become trending research because it can provide greater opportunity to obtain improvement in the polymer composite properties. Many researchers have proved that the use of a combined filler has further improved the mechanical, thermal, and physical properties of the polymer composite [36,82,83,84]. Often, the properties of the two fillers used as hybrid fillers are different and are able to have different effects on the polymer matrix used. Thus, this combination of fillers can result in the achievement of a variety of qualities that cannot be obtained by a single filler. According to Jaafar, the use of hybrid fillers will also affect the cost of composites, assist in acquiring specific properties of composites, and in turn improve the properties of the composites [85]. A cost reduction can be obtained when cheap and expensive materials are combined and used as a hybrid filler. However, there are still a limited number of studies involving dolomite as a co-filler in the hybrid filler system. Table 6 summarizes the use of dolomite as a co-filler/hybrid filler in the polymer composite system.

Saleh et al. proved that the use of dolomite as a co-filler in the epoxy resin can improve the properties of the epoxy/CNT composite [21]. They combined the organic (CNT) and inorganic filler (dolomite) in order to solve the problem of high agglomeration of the CNT. Therefore, the CNT and dolomite fillers were chemically attached using the chemical vapor deposition (CVD) method to allow the de-agglomeration of the CNT and assist in the dispersion of the hybrid filler in the epoxy matrix. They also used the physically hybrid method (PHY) for comparison with the CVD method. It is interesting to report that the epoxy with 5 wt% CNT/dolomite (CVD) composites is capable of increasing the tensile strength and tensile modulus of the epoxy by 67% and 28%, respectively, when compared to the virgin epoxy [21]. When using the PHY method, the tensile strength and tensile modulus of the epoxy/CNT/dolomite composite increased when the filler was added at 3% only. This shows that the CVD method performed better than the physically hybrid method. This was because the CNTs produced by the CVD method developed a preferable load transfer effect between the filler and the matrix with the presence of CNTs on the surface of dolomite, while in the physically hybrid method, the excess or unattached CNTs agglomerated and weakened the filler and matrix interfacial strength. This was proved by the image of the fractured surface of the epoxy/CNT/dolomite at 5% filler loading, where a homogenous dispersion was seen when using the CVD method, whereas the CNT agglomeration can be spotted when using the PHY method.

The work by Verma et al. involved the use of dolomite as a co-filler in the epoxy composite containing a natural fiber (Grewia optiva)/glass fiber hybrid filler. The epoxy composite had a fixed amount of Grewia optiva and glass fiber but with varying composition of the dolomite filler (0, 5, 10, and 15 wt%). Their findings indicate that the void content, density, hardness, and impact energy increase, while tensile and flexural strength decreases with the increase in the dolomite content [22,23]. At high loading, the dolomite agglomerated. This agglomeration is prone to intrinsic structural discontinuities that eventually deteriorate the tensile and flexural strength of the composite [22]. On the contrary, the hardness and impact strength of this epoxy hybrid composite system is reported to be increased. This might be attributed to the enhanced micro-packing of the ingredients in the polymer matrix. The stiffness properties of dolomite also helped to reduce the deformation characteristic of the epoxy matrix [23]. In another study, Verna et al. studied the effect of two different processing methods to produce an epoxy composite with hybrid fillers, which were hand layup and the VTRM technique. They found out that the use of both techniques may result in a decrement in the tensile strength of the epoxy, especially at high dolomite loading (15 wt%) [23]. However, by using the hand layup method, the tensile modulus, impact strength, and hardness of the epoxy can be increased even when the dolomite is added at 15 wt%.

Shamsuri et. al. investigated the mechanical and thermal properties of a low-density polyethylene/kenaf core fiber biocomposite with the addition of dolomite as a co-filler. They revealed that there is improvement in the tensile stress, tensile modulus, and impact strength of the low-density polyethylene (LDPE)/kenaf core fiber (KCF) composite as dolomite is added as the hybrid filler [24]. A significant increase in the tensile modulus and tensile stress of the LDPE/KCF was observed as the dolomite was added at 12 wt% to 18 wt%. The sliding effect of the KCF in the structure of LDPE might be hindered due to the rigid property of the dolomite, which can provide mechanical interlocking [24]. The impact strength of the LDPE/KCF/dolomite hybrid composite also increased with increasing of dolomite loading. At 18% of dolomite loading, the increment was reported to be 50.3% when compared to the virgin LDPE. In addition, the thermal properties of the LDPE were improved when a lower amount of dolomite was used (lower than 20%).

Md Saleh et al. have investigated the efficiency of the MWCNT/dolomite hybrid filler in enhancing the thermal conductivity of the phenolic resin. It was clearly observed that the hybrid MWCNT/dolomite filler assists the phenolic to achieve a higher thermal conductivity. The thermal conductivity value increased by 7.21% compared to that of the composite with a single filler [25]. The improvement of thermal conductivity was due to the synergistic effect of the dolomite and MWCNTs, which provides an additional channel for the heat flow to bypass the polymer matrix. Interestingly, the use of the hybrid filler also resulted in the improvement of the micro-hardness of the phenolic. The micro-hardness is also improved with increasing of dolomite loading. When added at 5%, the increment was reported to be the highest (+108%) [25]. In another study by Md Saleh et al. (2016), carbon nanotubes (CNTs) and dolomite were used as a hybrid filler in the phenolic composite [26]. Again, they studied the thermal conductivity and micro-hardness of the phenolic composite with a hybrid filler with a different preparation method (CVD and PHY method). In agreement to the previous study, the thermal conductivity and micro-hardness of the phenolic was successfully improved with the addition of dolomite and CNT as a hybrid filler. The improvement in hardness might be due to the decrease in inter-particle spacing with the presence of stiffer dolomite particles [86]. Good distribution of CNTs and dolomite in the phenolic composite also contributed to this enhancement [26]. Thus, a better conductivity pathway was created, while the hardness of the dolomite improved the micro-hardness of the phenolic composite [26].

Recently, Ni et al. performed a study on the use of a fibrous palygorskite (PAL)/dolomite (DOL) hybrid filler in the waterborne polyurethane (WPU) composite and its effect on the physical and mechanical properties of the WPU [27]. The results indicated that the addition of both fillers enhanced the mechanical and thermal properties of the WPU, but this was not happening when only a single filler (either one of the fillers) was used. The tensile strength of the WPU was at the highest value when a single filler of PAL and DOL was at 4% and 6% weight loading [27]. Interestingly, at 10% hybrid filler loading, the tensile strength could still be increased dramatically, by 178%, when compared to the neat WPU. This improvement was achieved because of the synergistic reinforcement effect of the two fillers used in the waterborne polyurethane polymer. Commonly, at higher filler loading, the tensile strength would decrease due to the agglomeration of the filler. Ni et al. proved through their SEM analysis that the hybrid fillers could be better dispersed inside the matrix when compared to the single filler. Obviously, when added in single form, both fillers are separated from the WPU matrix. This is one of the factors that contributed to the enhancement in the mechanical and thermal properties of the WPU composite.

Research by Özdemir et al. focused on the use of wood flour and dolomite as a hybrid filler in the polypropylene composite [28]. The composite samples were prepared by using a single-screw extruder. The research indicates that improvement in the tensile and flexural modulus of the PP can be realized when wood flour and dolomite are used as a hybrid filler. The addition of dolomite allowed an efficient stress transfer from the polymer matrix to the filler. The improvement in the tensile modulus can also be explained by the fact that the dolomite is much more rigid than the polymers and, thus, will stiffen the polymer matrix when it is embedded in the matrix structure [59].

Lastly, the work by Amiri et al. indicates the benefit of using a hybrid filler in enhancing the flame-retardant property of the polymer composite. They found out that the addition of a nanoclay and dolomite hybrid filler to polyurethane (PU) had a positive effect by way of reducing the flammability of the PU. Dolomite has a flame-retardant property, while the exfoliated nanoclay contains clay plates that act as a heat barrier and control the flame spread. The combination of both fillers resulted in a greater flame retardancy effect in the host polymer [29].

Based on the literature studies summarized in Table 6, it is worth mentioning that dolomite can play an important role in further improving the properties of the composite when used as a co-filler. It can help improve certain properties of the polymers that may not be achievable if only a single filler is used, properties such as hardness, impact strength, thermal stability, and flame-retardancy. In addition, dolomite is frequently used as a co-filler with carbon nanotubes and fibers, such as glass fiber and natural fiber. The differences in the physical and chemical characteristics between dolomite and those fillers can bring a more significant synergistic effect in enhancing the performance of the polymeric matrices.

Other than the above-explained research, there is also a study that indicated that the performance of a polymer is not necessarily improved through the addition of dolomite as a co-filler/hybrid filler. Vijayaraghavan et al. found out that the properties of polyurethane-based concrete did not improve with the addition of dolomite and kaolin as a hybrid filler [87]. This was due to the weak bond strength between the polymer and the hybrid filler. According to the results, the authors suggested that the ratio between the two fillers should be controlled and optimized. This is because the use of a hybrid filler will involve not only a matrix–matrix network, a matrix–filler network, and a filler–filler network but also a co-filler–co-filler network, a filler–co-filler network, and a filler–co-filler–matrix network. Sufficient network interactions between the matrix phase and both fillers (hybrid fillers) may attribute to the synergistic effect of fillers and the matrix, therefore leading to significant reinforcement of the polymer matrix.

## 4. Summary

This review article highlights the promising properties of the polymer composites containing a natural, inorganic, and mineral-based filler, which is dolomite. Here, we have summarized several findings based on the investigation of the polymer/dolomite composite system. Generally, the use of dolomite as a filler can produce polymer composite products with many beneficial properties. The dolomite filler can improve the hardness of polymeric matrices, even though no physical or chemical modification has been performed on it. The majority of the research indicates that the tensile strength, elongation at break, and Young’s modulus of polymers (either thermoplastic or thermoset) can be improved when incorporated with a physically modified dolomite filler. In this case, the physical modification of dolomite refers to the size reduction of dolomite using several techniques, such as high-energy milling, ultrasonication/tip-sonication, and grinding. Commonly, sieving of dolomite was performed to obtain dolomite with a uniform particle size. Chemical modification of dolomite was carried out by using silane treatment and stearic acid treatment and by the addition of a compatibilizer and a stabilizer. This chemical modification was generally performed to improve the compatibility of dolomite with the polymer matrices so that the mechanical and physical performance of the polymers can be enhanced. Furthermore, dolomite is in good demand for use as a co-filler in the hybrid polymer composite system. The results of adding dolomite as a co-filler in enhancing the physical and mechanical properties of the polymer composite are quite impressive, where it can improve certain polymer properties that may not be achievable if only a single filler is used, properties such as hardness, impact strength, thermal stability, and flame-retardancy.

The use of a polymer/dolomite composite will provide advantages to various industries, especially the industries that promote polymer composites, such as automotive, aerospace, construction, and biomedical. However, there are still improvements to be made in order to achieve better mechanical, thermal, and physical properties of the polymer/dolomite composite. This is because incorporation of dolomite with a polymer is still a challenging process because the agglomeration of dolomite needs to be addressed and improved. More studies on the chemistry and physico-chemistry aspects of dolomite are needed in order to further enhance the performance of the polymer/dolomite composite system. An optimistic perspective would see further development of dolomite as a nanofiller in a polymer nanocomposite system. Size reduction of dolomite particles to the nano-size range should be performed to ease its dispersion in the polymeric matrices and thus enhance the performance of the polymers. Polymer/dolomite nanocomposites can be aimed for more advanced applications, such as absorbable materials, biomedical applications, tissue engineering, wound healing, and automotive applications.

## Figures and Tables

**Table 1:** The physical properties of dolomite and calcite.

Physical Properties	Dolomite [51]	Calcite [52]
Chemical composition	(CaMg(CO_3_)_2_)	CaCO_3_
Color	Colorless, white, pink, gray, brown, black	White, colorless, gray, red, green, blue, yellow, orange, brown
Streak	White	White
Cleavage	Perfect, rhombohedral, three directions	Perfect, rhombohedral, three directions
Mohs hardness	3.5–4	3
Specific gravity	2.8–2.9	2.7
Diagnostic properties	Rhombohedral cleavage	Rhombohedral cleavage
Crystal system	Hexagonal	Trigonal

**Table 2:** Summary of dolomite usage.

Field	Purpose
Agriculture	As fertilizer [53] To decrease the acidity of soil [54] To adjust magnesium concentration in soil [54]
Aquaculture	To reduce phosphorus [56]
Ceramics	As a source of lime [46] In kiln control [53]
Chemical	To make salts, e.g., magnesia [57]
Construction	In cement and concrete manufacturing [41,53,55] As flooring material [42,58] As road construction material [58,59]
Plastic for structural application	As a filler [10]
Paint	As a filler [60] As an aid in pigmentation [61]
Wastewater treatment	For copper ion adsorption [43]
Membrane application	As low-cost membranes or substrates [62]
Plastics	As a filler to improve the mechanical properties [12,13]
Pharmaceutical	As a supplementary source of calcium and magnesium [63] As an osmotic oral laxative [63]

**Table 3:** Summary of literature studies reporting the type, processing method, and properties of polymer composites with dolomite fillers.

Type of Polymer	Processing Method	Dolomite Loading	Tensile Strength (MPa)	Elongation at Break (%)	Young’s Modulus (MPa)	Compression Strength (kg/m^2^)	Impact Strength (J/m)	Hardness (HV)	Flexural Strength (MPa)	Flexural Modulus (MPa)	Ref.
Polyester	Manually stir	0%	49	N/A	700	N/A	N/A	8	N/A	N/A	[10]
		5%	22	N/A	500	N/A	N/A	5	N/A	N/A	
		10%	22.5	N/A	820	N/A	N/A	5.5	N/A	N/A	
		20%	24	N/A	1010	N/A	N/A	24.9	N/A	N/A	
		30%	19	N/A	1100	N/A	N/A	12	N/A	N/A	
		40%	11	N/A	690	N/A	N/A	6	N/A	N/A	
		50%	18	N/A	810	N/A	N/A	6.5	N/A	N/A	
Polyether-based Polyurethane	Casting	0%	30	85	N/A	7	N/A	N/A	N/A	N/A	[65]
		10%	29	62	N/A	8	N/A	N/A	N/A	N/A	
		20%	23	50	N/A	11.8	N/A	N/A	N/A	N/A	
		30%	17	30	N/A	12.2	N/A	N/A	N/A	N/A	
		45%	16	20	N/A	14.2	N/A	N/A	N/A	N/A	
Polypropylene (PP)	Extrusion (by a twin-screw extruder)	0%	19	12	650	N/A	103.87	N/A	25	700	[64]
		5%	17	13	610	N/A	65.83	N/A	23	900	
		10%	16.5	18	710	N/A	50.42	N/A	24	2200	
		15%	16.2	17.9	790	N/A	41.35	N/A	23.5	1300	

**Table 4:** Summary of literature studies reporting the type, processing methods, and properties of polymer composites with physically modified dolomite fillers.

Type of Polymer	Processing Method	Dolomite Loading	Tensile		Elongation at Break (%)	Young’s Modulus (MPa)	Impact Strength (MPa)	Flexural		Ref.
			Strength (MPa)	Toughness				Strength (MPa)	Modulus (MPa)	
Epoxidize natural rubber (ENR 50)	Two-roll mill	Sample 1 (<63 µm)								[11]
		0 phr	N/A	N/A	N/A	N/A	N/A	N/A	N/A	
		5 phr	9.97	N/A	1070.56	N/A	N/A	N/A	N/A	
		15 phr	11.81	N/A	1264.21	N/A	N/A	N/A	N/A	
		25 phr	9.92	N/A	1198.24	N/A	N/A	N/A	N/A	
		35 phr	8.22	N/A	1062.74	N/A	N/A	N/A	N/A	
		50 phr	7.29	N/A	998.56	N/A	N/A	N/A	N/A	
		Sample 2 (75–150 µm)								
		0 phr	N/A	N/A	N/A	N/A	N/A	N/A	N/A	
		5 phr	7.42	N/A	1019.75	N/A	N/A	N/A	N/A	
		15 phr	10.94	N/A	1175.21	N/A	N/A	N/A	N/A	
		25 phr	8.94	N/A	1074.31	N/A	N/A	N/A	N/A	
		35 phr	7.86	N/A	1012.23	N/A	N/A	N/A	N/A	
		50 phr	5.83	N/A	942.45	N/A	N/A	N/A	N/A	
Polyethylene vinyl acetate (PEVA)	Twin-screw extruder	0%	10.5	130	410	0.74	N/A	N/A	N/A	[69]
		1%	11	142	350	0.85	N/A	N/A	N/A	
		3%	12.9	175	270	0.94	N/A	N/A	N/A	
		5%	13.9	198	250	1.05	N/A	N/A	N/A	
Polyethylene vinyl acetate (PEVA)	Heated two-roll mill	0%	6.2	3600	900	1.0	N/A	N/A	N/A	[70]
		1%	7.8	5300	1180	1.1	N/A	N/A	N/A	
		3%	7.5	5100	1100	0.95	N/A	N/A	N/A	
		5%	7.2	5000	1090	0.8	N/A	N/A	N/A	
Polybutylene terephthalate (PBT)	Twin-screw extruder	0%	37	N/A	19	990	61	62	2100	[9]
		5%	40.1	N/A	13	1250	49	62.2	2400	
		10%	43	N/A	9	1300	31	68	2500	
		15%	41	N/A	8	1500	20	64	2700	
Standard Malaysian rubber (SMR L)	Two-roll mill	Sample 1 (<63 µm)								
		0 phr	N/A	N/A	N/A	N/A	N/A	N/A	N/A	[11]
		5 phr	11.92	N/A	1378.24	N/A	N/A	N/A	N/A	
		15 phr	13.43	N/A	1582.24	N/A	N/A	N/A	N/A	
		25 phr	11.73	N/A	1293.51	N/A	N/A	N/A	N/A	
		35 phr	10.57	N/A	1210.95	N/A	N/A	N/A	N/A	
		50 phr	7.88	N/A	1187.24	N/A	N/A	N/A	N/A	
		Sample 2 (75–150 µm)								
		0 phr	N/A	N/A	N/A	N/A	N/A	N/A	N/A	
		5 phr	10.23	N/A	1287.42	N/A	N/A	N/A	N/A	
		15 phr	12.16	N/A	1394.13	N/A	N/A	N/A	N/A	
		25 phr	10.38	N/A	1253.12	N/A	N/A	N/A	N/A	
		35 phr	9.71	N/A	1184.38	N/A	N/A	N/A	N/A	
		50 phr	7.88	N/A	991.13	N/A	N/A	N/A	N/A	
Recycled polypropylene (rPP)	Z-blade mixer	Sample 1 (63 µm)								[71]
		0%	9	N/A	4.4	N/A	N/A	N/A	N/A	
		10%	9.8	N/A	5.8	N/A	N/A	N/A	N/A	
		20%	10	N/A	6.2	N/A	N/A	N/A	N/A	
		30%	12	N/A	9	N/A	N/A	N/A	N/A	
		40%	11	N/A	5	N/A	N/A	N/A	N/A	
		50%	8.8	N/A	4	N/A	N/A	N/A	N/A	
		Sample 2 (300 µm)								
		0%	9	N/A	4.4	N/A	N/A	N/A	N/A	
		10%	10	N/A	6	N/A	N/A	N/A	N/A	
		20%	11	N/A	8.9	N/A	N/A	N/A	N/A	
		30%	12	N/A	13.9	N/A	N/A	N/A	N/A	
		40%	10.8	N/A	9	N/A	N/A	N/A	N/A	
		50%	9.8	N/A	6.2	N/A	N/A	N/A	N/A	
Thermoplastic starch (TPS)	Mechanical stir	Sample 1 (150 µm)								
		0%	2.64 ± 0.13	N/A	95.6 ± 1.8	7.10 ± 0.69	N/A	N/A	N/A	[12]
		1%	1.76 ± 0.12	N/A	126.13 ± 7.17	7.13 ± 0.23	N/A	N/A	N/A	
		2%	1.73 ± 0.04	N/A	100.57 ± 5.9	8.90 ± 1.06	N/A	N/A	N/A	
		3%	1.98 ± 0.24	N/A	94.7 ± 4.75	9.07 ± 1.06	N/A	N/A	N/A	
		4%	2.67 ± 0.02	N/A	85.67 ± 0.68	10.10 ± 0.1	N/A	N/A	N/A	
		5%	2.68 ± 0.07	N/A	66.37 ± 3.85	10.23 ± 0.99	N/A	N/A	N/A	
		Sample 2 (U-50 µm)								
		0%	2.64 ± 0.13	N/A	95.6 ± 1.8	7.10 ± 0.69	N/A	N/A	N/A	
		1%	1.88 ± 0.05	N/A	165.77 ± 0.76	10.43 ± 0.95	N/A	N/A	N/A	
		2%	1.87 ± 0.10	N/A	134.13 ± 7.89	10.87 ± 0.85	N/A	N/A	N/A	
		3%	2.74 ± 0.17	N/A	106.90 ± 1.08	11.27 ± 0.06	N/A	N/A	N/A	
		4%	3.06 ± 0.16	N/A	100.37 ± 4.21	12.67 ± 0.15	N/A	N/A	N/A	
		5%	3.61 ± 0.30	N/A	96.17 ± 4.26	13.30 ± 0.2	N/A	N/A	N/A	

**Table 5:** Summary of literature studies reporting the different methods used to chemically modify dolomite and the impact on the properties of the polymer/dolomite composites.

Type of Polymer	Treatment on Dolomite	Processing Method	Dolomite Loading	Tensile Strength	Break of Elongation (%)	Young’s Modulus (MPa)	Impact Strength (J/m)	Hardness (BHN)	Flexural Strength (N/mm^2^)	Ref.
Polyethylene vinyl acetate (PECoVA)	Modification of dolomite with stearic acid	Heated two-roll mill	0%	15.4 ± 2 (MPa)	1151 ± 47	1.7 ± 0.1	N/A	N/A	N/A	[13]
			1%	20.7 ± 1 (MPa)	1354 ± 68	1.9 ± 0.1	N/A	N/A	N/A	
			3%	22.1 ± 1 (MPa)	1413 ± 87	2.0 ± 0.1	N/A	N/A	N/A	
			5%	18.5 ± 1 (MPa)	1252 ± 34	1.8 ± 0.1	N/A	N/A	N/A	
Polypropylene	Modification of dolomite with stearic acid	Twin-screw extruder	0%	24 (MPa)	290	1300	N/A	N/A	N/A	[17]
			5%	30 (MPa)	210	1290	N/A	N/A	N/A	
			10%	28 (MPa)	200	1510	N/A	N/A	N/A	
			15%	27 (MPa)	180	1512	N/A	N/A	N/A	
			20%	26.5 (MPa)	151	1513	N/A	N/A	N/A	
			25%	26 (MPa)	50	1700	N/A	N/A	N/A	
Polypropylene	Maleic anhydride as a compatibilizer	Not mentioned	0%	5 (MPa)	6	N/A	N/A	9	5	[19]
			5%	7 (MPa)	5	N/A	N/A	10	6	
			10%	10 (MPa)	4.2	N/A	N/A	13	7	
			15%	14(MPa)	4	N/A	N/A	17	13	
			20%	16 (MPa)	3	N/A	N/A	30	18	
Polyvinyl chloride (PVC)	Dolomite + stabilizer	Internal mixer	0 phr	53.2 (N/m^2^)	3.4	2200	50	N/A	N/A	[20]
			5 phr	56.9(N/m^2^)	4.6	2357	57	N/A	N/A	
			10 phr	63 (N/m^2^)	4.7	2450	60	N/A	N/A	
			15 phr	55.4 (N/m^2^)	4.3	2550	54	N/A	N/A	
			20 phr	45.5 (N/m^2^)	4.2	2655	52	N/A	N/A	

**Table 6:** Summary of literature studies reporting the use of dolomite as a co-filler/hybrid filler in polymer composites.

Polymer Matrix	Primary Filler	Secondary Filler	Processing Method	Filler Loading	Tensile			Elongation at Break (%)	Impact Strength	Hardness	Flexural		Ref.
					Strength (MPa)	Modulus	Stress (MPa)				Strength (MPa)	Modulus (MPa)	
Epoxy resin	Carbon nanotubes (CNTs)	Dolomite	Mechanical stirrer	Chemical vapor deposition									[21]
				0%	20.89	961.31 (MPa)	N/A	N/A	N/A	N/A	N/A	N/A	
				1%	28.52	1152.87 (MPa)	N/A	N/A	N/A	N/A	N/A	N/A	
				3%	33.83	1188.1 (MPa)	N/A	N/A	N/A	N/A	N/A	N/A	
				5%	4.93	1216.5 (MPa)	N/A	N/A	N/A	N/A	N/A	N/A	
				Physically hybrid									
				0%	20.89	961.31 (MPa)	N/A	N/A	N/A	N/A	N/A	N/A	
				1%	26.71	1139 (MPa)	N/A	N/A	N/A	N/A	N/A	N/A	
				3%	29.97	1159.8 (MPa)	N/A	N/A	N/A	N/A	N/A	N/A	
				5%	27.94	1148.7 (MPa)	N/A	N/A	N/A	N/A	N/A	N/A	
Epoxy resin	Glass fiber	Dolomite	Hand layup	0%	59.8	N/A	N/A	N/A	2.4 (J)	102.12 (HRL)	72.3	N/A	[22]
				5%	52	N/A	N/A	N/A	2.7 (J)	107.27 (HRL)	62.8	N/A	
				10%	45.9	N/A	N/A	N/A	3.4 (J)	109.60 (HRL)	54.3	N/A	
				15%	42.8	N/A	N/A	N/A	3.8 (J)	112.26 (HRL)	48..8	N/A	
Epoxy resin	Glass fiber	Dolomite	Hand layup	0%	282.58 ± 12.96	2.32 ± 0.14 (GPa)	N/A	N/A	6.1 ± 0.37 (J)	101.32 ± 3.08 (HV)	285.3 ± 13.12	N/A	[23]
				5%	268.59 ± 9.43	2.94 ± 0.l5 (GPa)	N/A	N/A	7.2 ± 0.36 (J)	103.76 ± 2.89 (HV)	273 ± 9.65	N/A	
				10%	244.69 ± 7.80	3.26 ± 0.13 (GPa)	N/A	N/A	8.8 ± 0.35 (J)	105.64 ± 3.23 (HV)	262.8 ± 7.12	N/A	
				15%	227.6 ± 7.10	2.44 ± 0.10 (GPa)	N/A	N/A	10.2 ± 0.41 (J)	108.22 ± 2.3 (HV)	249.9 ± 7.24	N/A	
			VTRM	0%	374.46 ± 17.80	3.54 ± 0.21 (GPa)	N/A	N/A	8.1 ± 0.46 (J)	102.94 ± 3.18 (HV)	337.4 ± 14.25	N/A	
				5%	337.33 ± 10.80	4.15 ± 0.17 (GPa)	N/A	N/A	9.2 ± 0.37 (J)	106.3 ± 225 (HV)	331.8 ± 10.12	N/A	
				10%	293.05 ± 11.65	4.33 ± 0.22 (GPa)	N/A	N/A	10.6 ± 0.48 (J)	109.06 ± 3.08 (HV)	327.6 ± 12.38	N/A	
				15%	270.43 ± 7.12	4.02 ± 0.12 (GPa)	N/A	N/A	13.4 ± 0.39 (J)	112.08 ± 2.38 (HV)	319.9 ± 7.60	N/A	
Low-density polyethylene (LDPE)	Kenaf core fiber (KCF)	Dolomite	Internal mixer	0%	N/A	109.37 (MPa)	1.47	N/A	3.52 (kJ/m^2^)	N/A	N/A	N/A	[24]
				3%	N/A	116.72 (MPa)	1.50	N/A	3.73 (kJ/m^2^)	N/A	N/A	N/A	
				6%	N/A	122.52 (MPa)	1.53	N/A	3.82 (kJ/m^2^)	N/A	N/A	N/A	
				9%	N/A	123.48 (MPa)	1.60	N/A	4.03 (kJ/m^2^)	N/A	N/A	N/A	
				12%	N/A	124.50 (MPa)	1.81	N/A	4.11 (kJ/m^2^)	N/A	N/A	N/A	
				15%	N/A	131.30 (MPa)	1.86	N/A	4.69 (kJ/m^2^)	N/A	N/A	N/A	
				18%	N/A	143.46 (MPa)	1.91	N/A	5.29 (kJ/m^2^)	N/A	N/A	N/A	
Phenolic	Dolomite	Multiwalled carbon nanotubes (MWCNTs)	Ball milling machine	0%	N/A	N/A	N/A	N/A	N/A	25(H_R_)	N/A	N/A	[25]
				1%	N/A	N/A	N/A	N/A	N/A	36(H_R_)	N/A	N/A	
				3%	N/A	N/A	N/A	N/A	N/A	45(H_R_)	N/A	N/A	
				5%	N/A	N/A	N/A	N/A	N/A	52(H_R_)	N/A	N/A	
Phenolic	Dolomite	Carbon nanotubes (CNTs)	Mechanical stirrer	Chemical vapor deposition									[26]
				0	N/A	N/A	N/A	N/A	N/A	25	N/A	N/A	
				1	N/A	N/A	N/A	N/A	N/A	39	N/A	N/A	
				3	N/A	N/A	N/A	N/A	N/A	46	N/A	N/A	
				5	N/A	N/A	N/A	N/A	N/A	50	N/A	N/A	
				Physically hybrid									
				0	N/A	N/A	N/A	N/A	N/A	25	N/A	N/A	
				1	N/A	N/A	N/A	N/A	N/A	30	N/A	N/A	
				3	N/A	N/A	N/A	N/A	N/A	37	N/A	N/A	
				5	N/A	N/A	N/A	N/A	N/A	38	N/A	N/A	
Waterborne polyurethane (WPU)	Fibrous palygorskite (PAL)	Dolomite	Mechanical stirring	0%	4.9	N/A	N/A	9.4	N/A	N/A	N/A	N/A	[27]
				WPU/PAL10%	3.5	N/A	N/A	8.8	N/A	N/A	N/A	N/A	
				WPU/DOL 10%	4.	N/A	N/A	9.5	N/A	N/A	N/A	N/A	
				WPU/MIX 10%	8.	N/A	N/A	8.1	N/A	N/A	N/A	N/A	
Polypropylene (PP)	Wood flour	Dolomite	High-intensity mixer and single-screw extruder	0%	27.1	474 (MPa)	N/A	29.4	N/A	N/A	37.1	1013	[28]
				3%	24.8	615 (MPa)	N/A	20.1	N/A	N/A	42.4	1592	
				6%	24.4	620 (MPa)	N/A	22.2	N/A	N/A	41.0	1617	
				9%	22.8	627 (MPa)	N/A	19.4	N/A	N/A	41.8	1673	

## Data Availability

There are no linked research datasets for this submission. Data will be made available on request.
